# Combined EUS-Guided Abdominal Cavity Drainage and Cystogastrostomy for the Ruptured Pancreatic Pseudocyst

**DOI:** 10.1155/2013/785483

**Published:** 2013-03-03

**Authors:** Ge Nan, Sun Siyu, Liu Xiang, Wang Sheng, Wang Guoxin

**Affiliations:** Shengjing Hospital, China Medical University, No. 36 Sanhao Street, Shenyang, Liaoning Province 110004, China

## Abstract

*Background*. Endoscopic-Ultrasonography- (EUS-) guided puncture and drainage of pancreatic pseudocyst is currently one of the most widely accepted nonsurgical treatments. To date, this technique has only been used for pancreatic pseudocysts adhesive to the gastric wall. This study introduces the technique of EUS-guided pseudocyst drainage and additional EUS-guided peritoneal drainage for the ruptured pseudocyst. *Methods*. Transmural puncture and drainage of the cyst were performed with a 19 G needle, cystotome, and 10 Fr endoprosthesis. Intraperitoneal drainage was performed with a nasobiliary catheter when rupture of pseudocyst occurred. The entire procedure was guided by the echoendoscope. *Results*. A total of 21 patients, 8 men and 13 women, with a mean age of 36 years, were included in this prospective study. All of the pseudocysts were successfully drained by EUS. Peritoneal drainage was uneventfully performed in 4 patients. There were no severe complications. Complete pseudocyst resolution was established in all patients. *Conclusion*. The technique of EUS-guided transmural puncture and drainage, when combined with abdominal cavity drainage by a nasobiliary catheter, allows successful endoscopic management of pancreatic pseudocysts without adherence to gastric wall.

## 1. Introduction

Endoscopic-Ultrasonography- (EUS-) guided pancreatic pseudocyst puncture and drainage are a widely accepted nonsurgical intervention [1–5].

Recent advances in understanding of the pathophysiology of pancreatic pseudocysts (PPs) allow the selection of optimal candidates for minimally invasive treatment approaches [6]. To date, the EUS-guided drainage approach has been limited to those cysts adherence to gastric wall, where it has been proven to be safe and effective. EUS-guided drainage of cysts without adherence to gastric wall can cause cyst collection leakage or even rupture, and for these patients a transpapillary or other approach is usually chosen [7].

A review of the literature indicates that this paper is the first to demonstrate the use of combined EUS-guided pseudocyst drainage for ruptured pancreatic pseudocyst and to provide an evaluation of the safety and effectiveness of this method.

## 2. Materials and Methods

### 2.1. Patients

**Figure 1:**
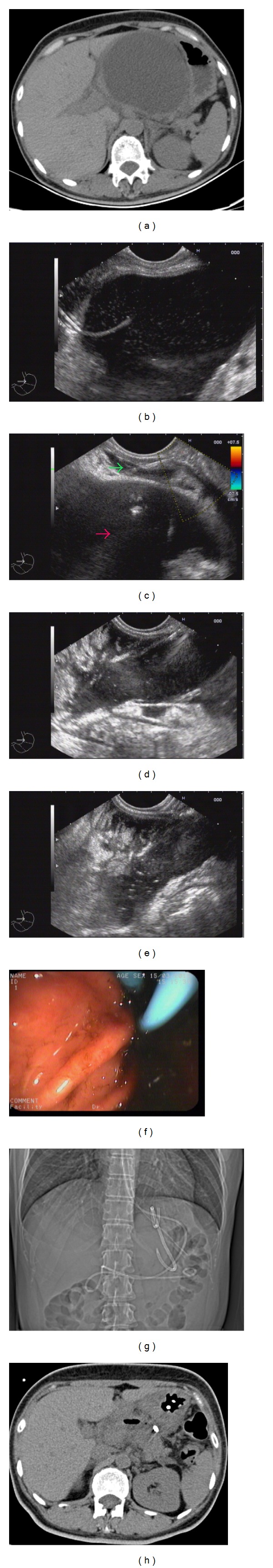
(a) CT shows a large cyst in the upper abdominal area. (b) EUS shows the cyst wall was 3 mm. The wall was not adhered to the gastric wall, as relative movement was observed. (c) After the needle puncture, cyst (red arrow) fluid will leak into the omental bursa. After cystotome dilation and stent placement, fluid leak (green arrow) begins to increase. (d) A large collection of fluid, measuring 3 cm, is seen below the cyst. (e) Transmural approach by a cystotome. (f) Intraperitoneal drainage by a 7 Fr nasobiliary catheter. (g) Drainage catheters seen on X-ray. (h) Pancreatic pseudocyst size is diminished, as confirmed by CT.

Inclusion criteria for this study are as follows: (1) pancreatic pseudocyst without adherence to gastric wall confirmed by CT and EUS; (Figures 1(a), and 1(b)) (2) pancreatic pseudocyst presenting with severe symptoms, such as abdominal pain, abdominal distension, duodenal obstruction, or biliary obstruction; (3) asymptomatic patient with pancreatic pseudocyst larger than 5 cm (considered a relative indication for drainage therapy in order to avoid serious complications, such as disruption or infection, in the future). Exclusion criteria were (1) thin, irregular pseudocyst wall; (2) coagulopathy; (3) unconfirmed diagnosis. All patients provided informed consent for the procedure. Complete blood counts, prothrombin time, and partial thromboplastin time were normal for all patients.

### 2.2. Devices

Longitudinal echoendoscope (PENTAX EG3830UT, Pentax Corporation, Japan) with a working channel of 3.8 mm accessible to a 10 Fr stent is used. Echo-Tip Ultra needle (19-G, Wilson-Cook Medic, USA) with a lumen of 0.8 mm in diameter is fitted to a 0.035 inch guidewire. Cystotome (10-Fr, Wilson-Cook Medic) is used to dilate the tract and create a large fistula. A nasobiliary drainage catheter (7-Fr, Wilson-Cook Medic) is used for peritoneal drainage or infected cyst drainage. A double pigtail stent (10 Fr, Endo-Flex GmbH, Germany) facilitates the cyst drainage.

### 2.3. Method

**Figure 2:**
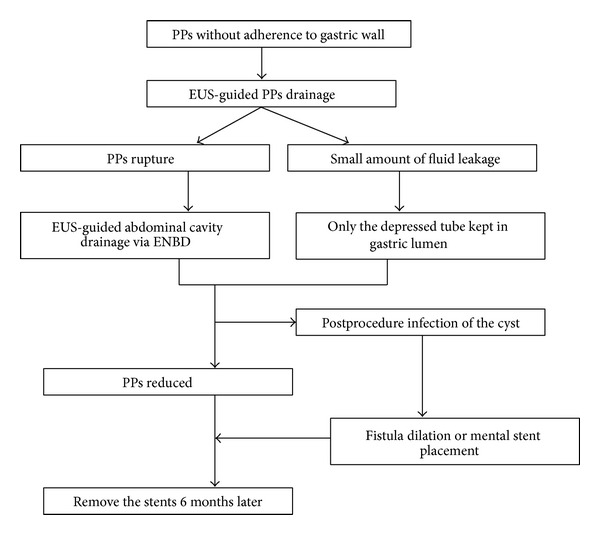
The patient candidate for our study is following the steps in chart.

The patient candidate for our study is following the steps shown in the chart in Figure 2. The echoendoscope-guided drainage procedure is described as below. The echoendoscope is introduced to scan for the pseudocyst and mark the puncture point. The contact zone (i.e., the closest approximation of the region between the gastric wall and the cyst wall) was identified. Color Doppler then is applied to identify the interposing vessels and thus avoid them during puncture. An Echo Tip Ultra endoscopic needle is then introduced via the working channel of the echoendoscope, and the cyst was punctured under EUS guidance. A sample of the cyst is aspirated for biochemical, cytological, and tumor marker analysis. If the cyst is very small, this sample should be limited to avoid rapid cyst deflation, which can cause increased difficulty during stent placement. The guidewire is inserted through the needle lumen into the cyst and coiled into 2-3 loops, and the needle is removed. The needle path is then dilated by the cystotome and a balloon dilator. A double pigtail (10 Fr) then is introduced for the drainage. The dilation is repeated when possible before placing stents to enhance efficacy.

If the cyst is ruptured with a large amount of fluid rush into the abdominal cavity, EUS-guided abdominal cavity drainage is introduced (Figures 1(c), 1(d), 1(e), 1(f), and 1(g)). The needle is again introduced via the working channel. Guided by EUS, the needle was punched into the abdominal cavity. The puncture site used in the cyst drainage procedure is preferentially considered. Guidewire was inserted through the needle lumen into the abdominal cavity. Deploy the 7 Fr nasobiliary drainage catheter via the guidewire. The nasobiliary drainage catheter with continuous aspiration was placed to complete the abdominal drainage. After the procedure, a tube remains in the stomach for decompression.

## 3. Results

A total of 86 patients with pancreatic pseudocyst treated at Shengjing hospital between May 2005 and June 2011 were enrolled in this study. 21 patients (13 women, 8 men) with pancreatic pseudocyst without adherence to gastric wall were selected for this procedure (Table 1).

All patients resumed regular diets after three days. Within one week of treatment, there was a reduction in cyst diameter of at least 50% in 19/21 patients, as measured by abdominal CT scan. Cysts in both of the patients in whom reduction of cyst diameter was less than 50% had an etiology of trauma.

4 patients had cyst ruptured, with intraperitoneal drainage kept for 3 days, and the gastric decompression tube for 2 days. No further infections were found in these patients.

Cyst infection was found in 2 patients in our study. EUS-guided secondary dilation of fistula was performed with an additional 10 Fr stent placement.

Stents were to be removed by endoscopy once cyst diameter was <3 cm, as measured by CT or EUS; this goal was achieved in 18 patients at 3-month followup. In the three remaining patients, stents were removed at six-month followup. At one year, no recurrence was found in any of the patients.

There were no severe procedure-related complications resulting from this technique; no bleeding, no perforation, no pneumoperitoneum. The postprocedure fever that developed in 2 patients was successfully managed by a secondary EUS-guided dilation. Results are reviewed in Table 2.

## 4. Discussion

A pancreatic pseudocyst is a collection of pancreatic fluid occurring within the pancreas or adjacent to it and surrounded by nonepithelialized tissue. It can occur after an episode of acute pancreatitis, trauma, or surgery, or in the setting of chronic pancreatitis. The cysts result from liquefaction of necrotic pancreatic tissue or from pancreatic duct obstruction or disruption [8]. Analysis of cyst fluid obtained during EUS should distinguish pancreatic pseudocyst from other cystic neoplasm. A high amylase or lipase content is typically seen in pseudocysts [9].

Management options available for pancreatic pseudocysts include endoscopic, radiologic (percutaneous), surgical (open surgery or Laparoscopic drainage), and conservative (medical) treatment [10]. The traditional treatment for pancreatic pseudocyst has been surgical, which has proven to be therapeutically effective, but is accompanied by high complication and mortality rates [11]. In recent years, there have been rapid gains in less invasive interventional techniques. CT and US-guided transcutaneous puncture and drainage have been widely applied. However, when the source of pancreatic pseudocyst is pancreatic fistula, simple aspiration therapy may result in recurrence rates of over 70% [12]. Transcutaneous external drainage may reduce this recurrence rate, but it can also greatly increase complication rates, from 5% to 60%, mainly due to perforation and hemorrhage [11]. Endoscope-guided transmural drainage is a recent intervention that provides continuous drainage via an endoprosthesis stent or a nasobiliary tube placed in a fistulous tract between the upper GI tract and the pseudocyst. This is only applied in cases of well-defined compression resulting from the cyst [13, 14]. If the cyst involves the gastric wall (e.g., the mucosa in the prominence emerges with a dark color or “Mosaic” sign), this treatment will be even more efficient. However, because it is a blind procedure, the risk of complication remains elevated. With the application of EUS guidance, blind puncture procedures should be phased out. Transpapilla drainage is another endoscopic treatment. Current consensus holds that cyst drainage through a stent placed in the pancreatic duct is insufficient because of the small lumen of the stent. Further, the long-term placement of a stent in the pancreatic duct is likely to induce morphological changes of the pancreatic duct and its surrounding tissues.

During the past decade, it has gradually been recognized that echoendoscopic treatment is a preferred approach in management of pancreatic pseudocysts [15–19]. Therefore, EUS-guided pancreatic pseudocyst drainage via cystoenterostomy should be considered as the first-line therapy [20]. With the addition of EUS guidance, selection of puncture points can be precise. As this minimally invasive technique matures, more patients should benefit from decreased trauma and fewer complications.

Pseudocysts may be classified according to anatomic location in relation to the omental bursa. Pseudocysts inside the omental bursa often have a common wall with the GI tract, and retroperitoneal perforation is rare when there is close apposition of the pseudocyst to the gastric wall. EUS-guided cystoenterostomy in this type of cyst usually has a low risk of complications and short recovery period. Over the past ten years, there have been numerous reports of successful treatment by this method, and it has become the recommended therapy for these cysts.

The other type of pseudocyst is located without adherence to gastric wall. This type of cyst usually has a wall that is separate from the gastric wall. Relative motion between the cyst wall and the gastric wall may be seen during EUS, particularly if the patient is instructed to take a deep breath. These cysts may be situated 2 cm or further from the GI wall as shown in our study. A transmural approach may cause cyst rupture or large leak of cyst fluid, resulting in ascites or infection. We think adequate drainage was effective to reduce the risk of infection. So, needle path dilation by cystotome or balloon was needed even they may have the higher risk of cyst rupture. Simple EUS-guided drainage of these ruptured cysts is usually not adequate. In the past, drainage by transpapillary placement of a stent in the pancreatic duct was considered if the cyst wall was behind omental bursa, but, as noted above, this approach has limited efficacy due to the smaller diameter of the stent.

This study proposes that cysts without adherence to gastric wall can be safely and effectively drained by EUS-guided cystoenterostomy accompanied by nasobiliary tube drainage of the abdominal cavity. To demonstrate this, the technique and the results of the procedure in 21 patients have been reported.

For surgeons, abdominal drainage for peritoneal infection or fistula in the abdominal cavity has been a routine [21, 22]. In this study, this method is successfully adapted to allow the echoendoscopic transmural drainage of pancreatic pseudocysts outside the omental bursa. A search of the literature has not revealed any other reports of this technique. Therefore, placement of the 7 Fr nasobiliary drainage catheters continuous abdominal drainage following cyst puncture and cystoenterostomy is the cornerstone concept in this treatment. The catheter allows the direct drainage of the leaked fluid from the abdominal cavity and prevents peritonitis. No infection was found in our 4 patients that performed abdominal cavity drainage. All this cavity drainage catheters were kept in place for 3 days. When we observed that no fluid was found from the catheters, patients had no symptoms including fever, and CT scan 3 days after the procedure shows the disappear of the leakage; catheters were removed. Since this is the first pilot study about EUS-guided abdominal cavity drainage, further studies focus on the efficiency of this method and the time to remove the abdominal catheters is still needed.

The main complications expected of this therapy are hemorrhage and infection. Hemorrhage can be avoided by using color Doppler for the detection and avoidance of interposing vessels during the puncture. Some studies are considered performing the povidone-iodine washing of gastrointestinal mucosa to prevent the infection [23]. In our study antibiotic drugs were taken after the procedure to prevent the infection caused by the needle puncture. We only observed cyst infection in one patient. Cyst infection was successfully treated with a secondary EUS-guided dilation. Placement of covered metal stent with larger lumen can also be considered [19].

## 5. Conclusion

The technique of EUS-guided transmural drainage of pancreatic pseudocysts without adherence to gastric wall combined with drainage of the abdominal cavity by a nasobiliary catheter allows for successful endoscopic management with a low risk of complications. This should lead to expanded application of EUS-guided pancreatic pseudocyst drainage.

Results from larger series will be necessary to learn more about this procedure and its ultimate role in the treatment of pancreatic pseudocysts without adherence to gastric wall.

## Figures and Tables

**Table 1:** Patient characteristics in this study.

Patients details	
Patients, total	21
Male : Female	8 : 13
Age, mean, years (range)	36 (10–45)
Location of cyst	
Head	2
Body	18
Tail	1
The distance from the cyst to the gastric wall, cm	2.1 (1.5–3)
Diameter of cyst, cm	7.6 (7–10)
Cause of the cyst	
Trauma	3
Severe pancreatitis	16
Postoperative	2

**Table 2:** Patients results of EUS-guided cystogastrostomy.

Patient details	
Completely recovery	21
Cyst rupture during the procedure	4/21
Symptoms after EUS drainage	
Fever	1
Abdominal pain	0
Others	0
Decompression tube in place, days	2-3
Postoperative hospital stay, days	4–10 (4.3)
